# Newly Developed Vanadium-Based Glasses and Their Potential for Nuclear Radiation Shielding Aims: A Monte Carlo Study on Gamma Ray Attenuation Parameters

**DOI:** 10.3390/ma14143897

**Published:** 2021-07-13

**Authors:** Huseyin Ozan Tekin, Ghaida Bilal, Hesham M. H. Zakaly, Gokhan Kilic, Shams A. M. Issa, Emad M. Ahmed, Yasser Saad Rammah, Antoaneta Ene

**Affiliations:** 1 Department of Medical Diagnostic Imaging, College of Health Sciences, University of Sharjah, Sharjah 27272, United Arab Emirates; tekin765@gmail.com (H.O.T.); GBilal@gmail.com (G.B.); 2 Medical Radiation Research Center (USMERA), Uskudar University, Istanbul 34672, Turkey; 3 Center for Advanced Materials Research, Research Institute of Sciences and Engineering, University of Sharjah, Sharjah 27272, United Arab Emirates; 4 Institute of Physics and Technology, Ural Federal University, 620000 Ekaterinburg, Russia; 5 Physics Department, Faculty of Science, Al-Azhar University, Assiut 71524, Egypt; shams_issa@yahoo.com; 6 Department of Physics, Faculty of Science and Letters, Eskisehir Osmangazi University, Eskisehir 26040, Turkey; gkilic@ogu.edu.tr; 7 Physics Department, Faculty of Science, University of Tabuk, Tabuk 71451, Saudi Arabia; 8 Department of Physics, College of Science, Taif University, Taif 21944, Saudi Arabia; makboul67@yahoo.com; 9 Department of Physics, Faculty of Science, Menoufia University, Shebin El-Koom, Menoufia 32511, Egypt; dr_yasser1974@yahoo.com; 10 INPOLDE Research Center, Department of Chemistry, Physics and Environment, Faculty of Sciences and Environment, Dunarea de Jos University of Galati, 47 Domneasca Street, 800008 Galati, Romania

**Keywords:** Phy-x PSD, CuO-doped, radiation shielding, vanadate glasses

## Abstract

This study aimed to investigate different types of glasses based on the 46V_2_O_5_-46P_2_O_5_-(8-x) B_2_O_3_-xCuO system in terms of their nuclear radiation shielding properties. Accordingly, five different CuO-doped vanadate glasses were investigated extensively to determine the necessary gamma shielding parameters along with effective conductivity at 300,000 and buildup factors. Phy-x PSD software was used for determination of these vital parameters. Furthermore, these parameters, such as half value layer, tenth value layer, and mean free path were investigated in a broad energy range between 0.015 and 15 MeV. The results revealed that the amount of CuO reinforced in each sample plays an essential role in determination of the shielding abilities of the samples. The sample with the highest CuO content had the highest linear attenuation coefficient and mass attenuation coefficient values. Additionally, the lowest mean free path, half value layer, and tenth value layer values were recorded for glass sample VPCu8. There was an inverse relation between the effective conductivity and effective atomic number and photon energy; that is, as energy increases, the effective conductivity and effective atomic number decreased rapidly, especially in the regions of low energy. Glass sample VPCu8 reported the highest values for both parameters. Moreover, glass sample VPCu8 had the lowest exposure buildup factor and energy absorption buildup factor values. Our findings showed that CuO-reinforced vanadate glass composition, namely 46V_2_O_5_-46P_2_O_5_-8CuO, with a glass density of 2.9235 g/cm^3^, was reported to have superior gamma ray attenuation properties. These results would be helpful for scientists in determining the most appropriate additive rare earth type, as well as the most appropriate glass composition, to offer shielding characteristics similar to those described above, taking into consideration the criteria for usage and the needs of the community. The results of this research will be useful to the scientific community in evaluating the prospective characteristics of CuO-doped glass systems and related glass compositions. CuO-doped glass systems and associated glass compositions have a wide range of properties.

## 1. Introduction

With the widespread use of radiation sources and radioactive materials in today’s world, it is more important than ever to use radiation sources carefully and safely. Concrete and lead are frequently used as radiation-shielding media; nevertheless, concrete has several drawbacks, including lack of transparency and heavy weight, to name a few, while the toxicity of lead-based radiation shielding systems, as well as their lack of structural integrity, are the most significant disadvantages. Consequently, radiation shielding systems must be developed that are corrosion resistant, biocompatible, and capable of being shaped into slim, compact designs with excellent structural integrity and endurance. Glass with the properties of transparency to visible light, ease of fabrication, and easy property adjustment through composition and preparation procedures proved to be effective shielding materials [1,2]. The unique features of vanadate glasses, as well as their prospective appropriateness for applications, continue to pique researcher’s curiosity. Vanadate-based glasses have garnered considerable attention in recent decades due to their low crystallization tendency, low melting point, excellent semiconducting properties, chemical resistance, and thermal conductivity, among other properties that make them an excellent material for a variety of applications. These glasses exhibit semiconducting properties due to the electron hopping behavior of vanadium ions between the V^4+^ and V^5+^ valence states. This has disadvantages for vanadium-based glasses, including decreased transparency. When PbO is added to any glass system, the structure of the glass is substantially altered. Due to their high electrical conductivity, stability, and wide glass formation range, vanadate glasses produced with PbO are especially interesting. There are a variety of advantages to phosphate-based glasses, including excellent UV transmittance, low melting and glass transition temperatures, and better thermal stability [3,4,5,6,7]. They may, however, be less resistant to chemical attack than borate glasses containing B_2_O_3_, one of the most often used glass formers. Borate-based glasses are well-suited for constructing novel optical devices due to their high solubility in rare earth ions, simplicity of mass production, and cheap cost. B_2_O_3_ glass has a lower melting point and a greater transmittance value. By doping borate glasses with transition metal ions, it is possible to enhance their optical transmittances, electrical conductivities, thermal conductivities, and even magnetic properties. Notably, glass systems containing B_2_O_3_ exhibit excellent broad band characteristics and exhibit significant increases in luminescence intensities and lifetimes. PbO may be employed as a network modifier or a network former in the glass network, depending on its composition. Additionally, significant quantities of it may be accommodated in oxide glasses. The electrical conductivity, stability, and wide glass forming range of vanadate glasses produced with PbO make them especially attractive. When vanadium is coupled with a stronger glass former, such as B_2_O_3_ or P_2_O_5_, a more stable network can be created [7,8,9,10,11]. Copper, as is well known, has a broad selection of applications due to factors such as being incomparable in terms of electrical conductivity after silver among all metals. Because of its thermal and corrosion resilience, it is a significant transition metal oxide to use in applications ranging from industrial applications to use as nuclear shields. CuO-doped glasses are also gaining popularity because of their semiconducting characteristics [5]. On this basis, the aim of the novel study is to use MCNPX (version 2.7.0, Los Alamos National Laboratory, Los Alamos, New Mexic) [12] general-purpose Monte Carlo code and Phy-X/PSD software (Adobe Inc., Mountain View, CA, USA) [13] to investigate the nuclear shielding properties of glass samples from the 46V_2_O_5_-46P_2_O_5_-(8-x)B_2_O_3_-xCuO system in a wide range of energies between 0.015 and 15 MeV. The findings of this work will be valuable in this subject within the glass literature, particularly with respect to radiation shielding. We want to offer some future research for the scientific community to conduct as part of their continued work on this currently promising glass system. In this study, we provide thorough findings based on a variety of factors. However, given the substantial material qualities of glass, it may be argued that continued work is necessary to optimize and enhance the suggested glassy system. As a result of the outcomes of this investigation, researchers will be able to learn more about the usefulness of CuO-doped glasses as nuclear shielding materials.

## 2. Materials and Methods

**Figure 1:**
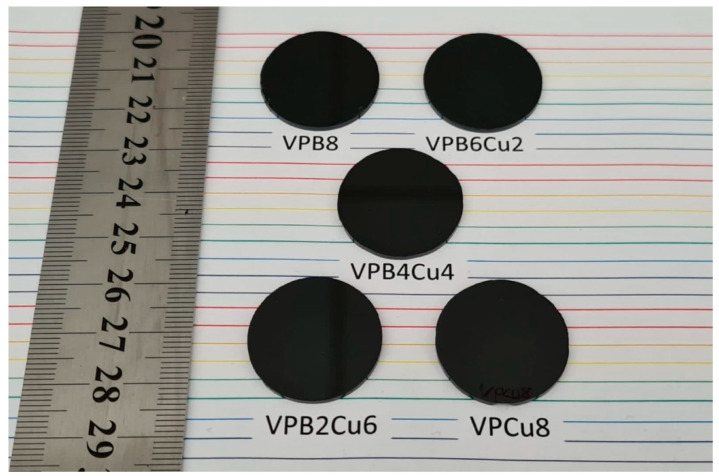
Physical appearances of Cu-doped VPB_x_Cu_y_ glasses along with their codes.

Five distinct CuO-doped VPB glass samples [5] were thoroughly studied in this study in terms of their nuclear attenuation shielding characteristics, effective conductivity, and accumulation factors. Figure 1 shows the physical appearances of Cu-doped VPB_x_Cu_y_ glasses along with their codes The chemical properties of the glass samples under examination can be listed as follows:

1. 46V_2_O_5_-46P_2_O_5_-8B_2_O_3_
2. 46V_2_O_5_-46P_2_O_5_-6B_2_O_3_-2CuO
3. 46V_2_O_5_-46P_2_O_5_-4B_2_O_3_-4CuO
4. 46V_2_O_5_-46P_2_O_5_-2B_2_O_3_-6CuO
5. 46V_2_O_5_-46P_2_O_5_-8CuO

In the reference study [5], the densities of the glass samples were reported as 2.8123, 2.8429, 2.8656, 2.8984, and 2.9235 g/cm^3^ for the samples VPB8, VPB6Cu2, VPB4Cu4, VPB2Cu6, and VPCu8, respectively (see Table 1). The determined nuclear radiation shielding parameters, as well as technical details, will be presented in this section. A brief overview of the Phy-x PSD calculate will also be presented.

### 2.1. Nuclear Radiation Shielding Properties

It is necessary to comprehend the Lambert-Beer law, shown in Equation (1), to simplify the mass attenuation coefficient.

(1) $$I=I_{0}e^{-\mu t}$$

The equation illustrates the relationship between the initial radiation intensity (*I*_0_), the radiation intensity after passing through the absorber (*I*), the absorber thickness (*t*), and the linear radiation attenuation coefficient (*μ*). 

The mass attenuation coefficient (*μ*_m_) [14,15] is a measurement of the likelihood of incident photons to interact with matter per unit density. It is calculated using the formula demonstrated in Equation (2):

(2) $$\mu_{m}=\frac{\mu }{\rho }$$

where *μ*: linear attenuation coefficient and *ρ*: density of the glass sample.

The values of *μ*_m_ were calculated using the Equation (3):

(3) μm=∑iwi(μρ)i

where (*μ*_m_) is the mass attenuation coefficient, (*w_i_*) is the weight fraction of the *i*th constituent element, (*μ*) is the linear attenuation coefficient, and (*ρ*) is the density. The mean free path (MFP) is the mean distance traveled by a photon before it interacts with a shielding substance. Equation (4) is used to determine this:

(4) $$\mathrm{MFP}=\frac{1}{\mu }$$

The power of monoenergetic gamma rays is lowered by approximately 37% after traversing 1 MFP through an attenuator environment, in an ideal narrow-beam geometry. A dimensionless quantity is produced by multiplying the linear attenuation coefficient by the distance in centimeters between the point source and the detector and this dimensionless quantity is termed as optical thickness (OT). The OT illustrates how many MFP lengths the gamma-photons completed as they transverse the shield [16]. The half value layer (HVL) is the thickness of the absorber necessary to reduce the radiation intensity to 50% of its original value. It is computed using the following equation:

(5) $$\mathrm{HVL}=\frac{ln\;\left(2\right)}{\mu }$$

As with HVL [17,18], the tenth value layer (TVL) is defined as the thickness of the absorber required to lower the radiation intensity to one tenth of its original value. It is computed using the following Equation (6):

(6) $$\mathrm{TVL}=\frac{ln\;\left(10\right)}{\mu }$$

The following Equation (7) relates the effective conductivity C_eff_ (s/m) of a shielding material for attenuation at 300 K to the effective number of electrons per-gram N_eff_ (electrons/g):

(7) $$C_{\mathrm{eff}}=\left(\frac{N_{\mathrm{eff}}\;\rho \;e2\;\tau }{m_{e}}\right)\times 10^{3}$$

where *ρ*, *e* and *m_e_* denote the density of shielding materials (g/cm^3^), the charge on an electron (C), and the electron’s rest mass (kg), respectively. *τ* depicts the electron’s relaxation time and is calculated using the following Equation (8):

(8) $$\tau =\frac{h}{600\pi k}$$

where h: Planck’s constant and k: Boltzmann constant.

The effective atomic number is a parameter that describes how multi-element structures respond to ionizing radiation. The direct method was used in this study to determine the effective atomic number by evaluating the atomic and electronic cross sections and is calculated using Equation (9):

(9) $$Z_{\mathrm{eff}}=\frac{\Sigma_{i}f_{i}A_{i}{\left(\frac{\mu }{\rho }\right)}_{i}}{\Sigma_{j}f_{j}\left(\frac{A{}_{j}}{Z_{j}}\right){\left(\frac{\mu }{\rho }\right)}_{j}}$$

where *f_i_*, *A_i_*, *Z_j_*: fraction by mole, atomic weight, and atomic number of *i*th constituent element, respectively. Incoherent scattering is used exclusively to determine a shielding material’s equivalent atomic number (Z_eq_). Buildup factor (BUF) computations are made easier with Z_eq_ values.

Z_eq_ values in this study were obtained using the interpolation method, demonstrated in Equation (10):

(10) $$Z_{\mathrm{eq}}=\frac{Z_{1}\left(logR_{2}-logR\right)+Z_{2}\left(logR-logR_{1}\right)}{logR_{2}-logR_{1}}$$

where the ratio *R* is the defining factor for the equivalent atomic number for distinct photon energy.

(11) $$R=\frac{\mu_{m\;compton}\;}{\mu_{m\;total}}$$

*Z*_1_ and *Z*_2_ are the elements’ atomic numbers, which correspond to their respective ratios *R_1_* and *R_2_*, respectively. Photons that pass through the body lose energy and are progressively absorbed, but they also scatter numerous times, producing new photons. The ratio of the total number of photons to the ratio of unscattered photons is a simple definition of a buildup factor. The ANS-standard was created to calculate gamma ray BUFs for a point isotropic source operating at a range of energies between 0.015 and 15 MeV. The energy absorption buildup factor (EABF) is concerned with the amount of energy absorbed or retained by the interacting material. EABFs are frequently logged using the geometric progression (G-P) fitting method. After determining the Z_eq_ values, the five G-P fitting parameters for the elements (b, a, c, and d Xk) are obtained from the ANS-standard database, which contains a variety of elements with energies ranging from 0.015–15 MeV to 40 mfp. The interpolation method was used to determine the G-P fitting parameters for the glass materials. The following formulas (10)–(14) are used to calculate the energy absorption buildup factor (EABF), the exposure buildup factor (EBF), and photon-dose multiplication factor (K) for a single-layered gamma ray shielding enclosure (GSE) with an OT of up to 100 mfp and an energy range of 0.015 to 15 MeV.

(12) $$B\left(E,X\right)=1+\frac{b-1}{K-1}\;\left(K^{x}-1\right)\;for\;K\;\neq \;1\;$$

(13) B(E,X)=1+(b−1)x for K=1

(14) $$K\left(E,x\right)=cx^{a}+\frac{d\;\tan h\frac{x}{X_{k}-2}-\tanh\left(-2\right)1-\tan h\left(-2\right)}{1-\tan h\left(-2\right)}\;for\;x\;\leq 40\;mfp$$

The abbreviation in the formulas can be listed as follows.

- *E*: Incident photon energy
- *X*: Penetration depth in mfp
- *B*: Buildup factor at 1 mfp
- *K*: Photon-dose multiplication factor [19].

### 2.2. Phy-x Photon Shielding and Dosimetry (PSD)

This user-friendly web application calculates shielding and dosimetry values [13]. The first stage in obtaining findings is to carefully determine the composition of the material that will be used in the calculations. The material composition can be inputted into the software in two ways: as a mole fraction or as a weight fraction; elemental weight fractions were used for the purpose of this study. Each sample’s density (g/cm^3^) is then input. Two energy zones were set in the program: 15 keV–15 MeV and 1 keV–100 GeV. In this study, shielding parameters were computed at energies ranging from 15 keV to 15 MeV. The user then selects the number of parameters to be determined. Following the completion of the preceding stages, the software calculates LAC-Linear attenuation coefficient, MAC-Mass attenuation coefficient, HVL-Half Value layer, TVL-Tenth value layer, C_eff_- effective conductivity, and other parameters and arranges them in an easily understandable Excel sheet. Furthermore, the most common radiation shielding parameters are compared so that users can analyze the study results more precisely. The most important feature of this program is that it can calculate all of the shielding and dosimetry parameters specified above quickly and correctly for an endless number of distinct samples. This program is intended to help researchers produce low-cost, long-lasting shielding materials by allowing them to do accurate shielding calculations in a short amount of time.

### 2.3. Monte Carlo Simulations (MCNPX Version 2.7.0)

The mass attenuation coefficients of the VPB_x_Cu_y_ glasses were efficiently estimated using the general-purpose Monte Carlo algorithm MCNPX (version 2.7.0 Advanced Accelerator Applications Los Alamos National Laboratory, Los Alamos, NM, USA). To begin, input data for MCNPX was created using the basic components as follows:

- Cell card
- Surface card
- Source information

**Figure 2:**
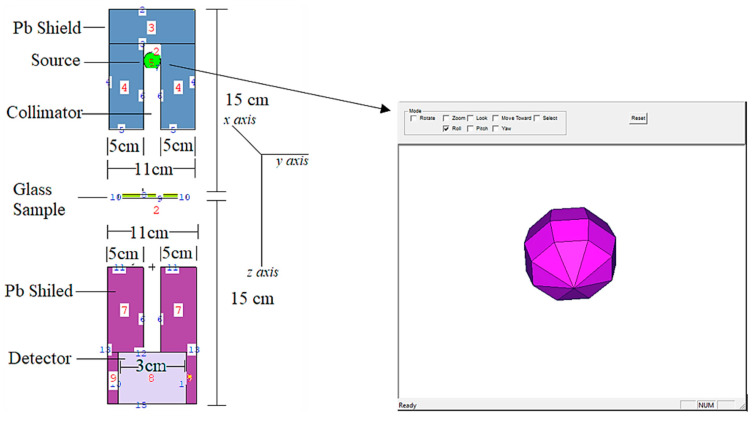
2-D view and dimensions of designed MCNPX simulation setup for gamma ray transmission competencies of VPB_x_Cu_y_ glasses (obtained from MCNPX Visual Editor VE VisedX22S).

A point isotropic gamma ray source was housed inside a lead (Pb) shield block for radiation protection (see Figure 2).

**Figure 3:**
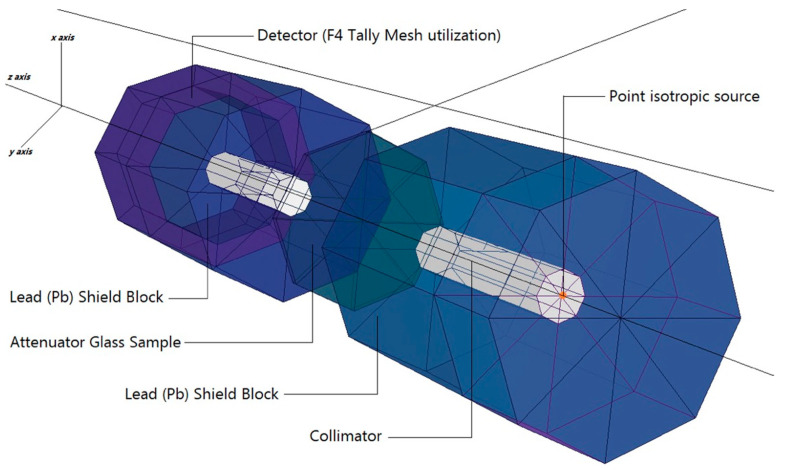
3-D view of designed MCNPX simulation setup for gamma ray transmission competencies of VPB_x_Cu_y_ glasses (obtained from MCNPX Visual Editor VE VisedX22S).

As a result, models of the VPB_x_Cu_y_ glass specimens were created based on their elemental compositions (in percent weight) and material densities (in grams per cubic centimeter) and were then tested. The glass specimen had a radius of 5 cm and a cylindrical form. As a result, the material characteristics needed for cell cards were included in the card’s border design (i.e., elemental mass fraction and material density). Illustration of a two-dimensional (2-D) perspective and dimensions of the proposed MCNPX simulation setup for testing the gamma ray transmission capabilities of VPB_x_Cu_y_ glasses is shown in Figure 2 (obtained from MCNPX Visual Editor VE VisedX22S). The modeled point isotropic source may alternatively be viewed as an extension of the overall gamma ray transmission arrangement in Figure 2. The elemental mass fractions of the fabricated VPB_x_Cu_y_ glasses are listed in Table 1. It should be noted that the Mn variable was used in the MCNPX INPUT file to indicate the elemental composition of the glass specimens, which should be recorded. After completing the first cell description process, it was determined that photon and electron interactions (i.e., IMP: p, e) were significant. The MCNPX code, which implements a variance reduction technique, may be regarded as an example of such a method. However, on the opposite side of the VPB_x_Cu_y_ glass material, a detector field (F4 Tally Mesh, KnitMesh Technologies Ltd., Greenfield, UK) was installed for the purpose of counting attenuated gamma rays. In order to determine the photon flux on an average basis at a point or in a cell, this kind of tally mesh may be used to assist. In all, 10^8^ particles with different photon energies were used in each glass sample, with each run being performed twice (i.e., from 0.015 MeV to 15 MeV). Overall, MCNPX had an uncertainty of less than 1%. Finally, Figure 3 shows the 3-D view of designed MCNPX simulation setup for gamma ray transmission competencies of VPB_x_Cu_y_ glasses (obtained from MCNPX Visual Editor VE VisedX22S). As can be observed in Figure 3, the beam axis of primary gamma ray was set on the *z*-axis.

## 3. Results and Discussion

In this study, the gamma ray attenuation capabilities of five different glass samples from the 46V_2_O_5_-46P_2_O_5_-(8-x) B_2_O_3_-xCuO system were extensively investigated. To verify the obtained results from MCNPX and Phy-X/PSD, the mass attenuation coefficients of VPB_x_Cu_y_ glasses were compared in terms of their statistical differences (see Equation (14)) at each gamma ray energy.

(15) $$∆\;\left(\%\right)=\frac{\left|\mu_{m}\left(\mathrm{MCNPX}\right)-\mu_{m}\left(\mathrm{Phy-X/PSD}\right)\right|}{\left[\mu_{m}\left(\mathrm{MCNPX}\right)+\mu_{m}\left(\mathrm{Phy-X/PSD}\right)\right]/2}\times 100$$

The obtained mass attenuation coefficients, along with their relative deviations, are presented in Table 2. As a result of this comparison, a good agreement is reported between MCNPX and Phy-X/PSD. According to the tools used, namely MCNPX and Phy-X/PSD, it can be said that the corresponding mass attenuation coefficient values of VPB_x_Cu_y_ are theoretically valid. However, slight differences at lower energies were also reported. This can be considered a normal situation, since the working principles and measurement flows of those two platforms are totally different. On one hand, Phy-X/PSD is a platform that can calculate the mass attenuation coefficients of shielding materials considering their pre-defined elemental mass fractions (wt.%) and densities (g/cm^3^). Moreover, there are no required definitions for equipment of gamma ray transmission setup. On the other hand, MCNPX is a typical radiation transport code, which requires the definition of entire setup including source, energy, material shape, material properties, collimators, and detectors in the input (INP) file. In short, a user should define the gamma ray transmission setup in consideration of the actual properties of the mentioned tools in MCNPX. However, we used the verified coefficients for the determination of further parameters (see Section 2.1).

**Figure 4:**
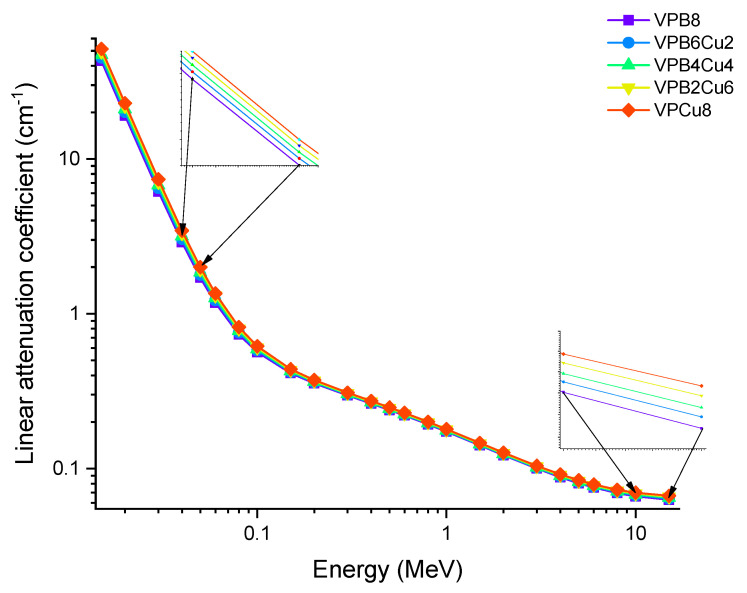
Variation of linear attenuation coefficient (*μ*) against photon energy for VPB_x_Cu_y_ glass samples.

**Figure 5:**
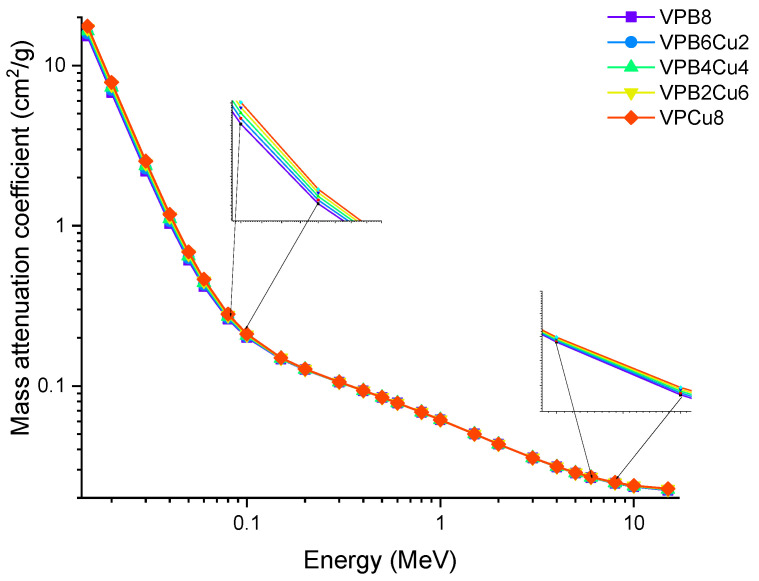
Variation of mass attenuation coefficient (*μ*_m_) against photon energy for VPB_x_Cu_y_ glass samples.

Photon energy has a significant effect on the linear attenuation coefficient (*μ*) of the investigated glass samples, as shown in Figure 4 (from 0.015 MeV to 15 MeV). To get energy, photons may do so by one of two methods, both of which include the usage of electrons. For example, in one instance, a photon is fully devoured; in another, just a portion of the photons is absorbed, leaving the other photons dispersed. The photoelectric effect seems to be greater for low-energy photons (0.1 MeV) than for high-energy photons (0.8 MeV). As the Z number increases, the likelihood of this interaction occurring increases dramatically as well [20]. The Compton effect is most noticeable when photons have a medium to high energy (>0.1 MeV). Pair formation happens when photons have an energy higher than 1.02 MeV and collide with one another. Because of these kinds of interactions, it is reasonable to infer that the linear attenuation coefficient is energy dependent, and that it changes according to the photon energy regions in which it occurs. A rapid decrease in the LAC values was observed in Figure 4, between 0.015 and 0.08 MeV, where the photoelectric effect is dominant. As with the first region in the graph, a smooth decrease was observed in the second region pertaining to the superiority of Compton scattering. The results indicate that LAC values were highest for the VPB8 glass sample. This can be justified by the amount of CuO enforced in the sample. As seen in Table 1, VPCu8 has the highest density (i.e., 2.9235 g/cm^3^) and has the highest percentage of CuO when compared to the other samples. The increase in CuO concentration in the glass samples resulted in a substantial increase in density as well. The sample with 0% Cu (i.e., VPB8) has the lowest density (2.8123 g/cm^3^), whereby the sample with 8% CuO has the highest density (2.9235 g/cm^3^). The aforementioned situation is due to the higher atomic number of Cu (Z = 29). The fluctuation of the mass attenuation coefficient (*μ*_m_) as a function of photon energy is seen in Figure 5.

**Figure 6:**
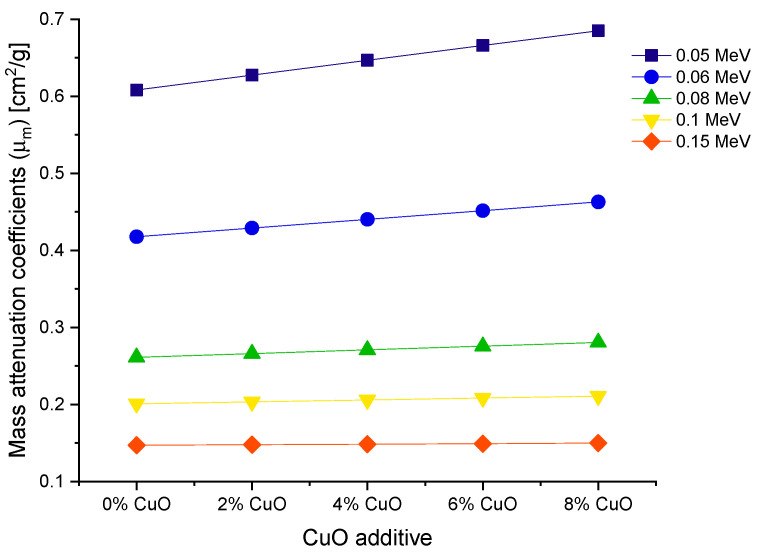
Variation of mass attenuation coefficients as a function of increasing CuO reinforcement.

Instead of a distance, the attenuation rate could be expressed in terms of the mass of the object that photons encounter. The area mass of an attenuator, not its overall mass, is the factor that governs its attenuation rate [21,22,23]. The best explanation for this occurrence is in terms of mass attenuation coefficients (*μ*_m_). The *μ*_m_ is a density-dependent parameter for assessing a material’s shielding abilities. In this study, the samples’ *μ*_m_ values were estimated in the photon energy ranges of 0.015 and 15 MeV. Figure 5 shows the distribution of *μ*_m_ as a function of incident photon energy for all glass samples. A similar trend to that seen in LAC values is observed. In *μ*_m_ values, the preponderance of the aforementioned interactions was also reported. The gradual fluctuations in the density of glass samples are responsible for this similarity. Because values of *μ*_m_ may be obtained by dividing density by LAC, a similar trend is expected. Additionally, VPCu8 has the highest *μ*_m_ values. This could be due to the sample’s overall mass being directly affected by the Z number and its increase from VPB8 to VPCu8. A detailed analysis was performed to obtain the changes in mass attenuation coefficients as a function of increasing CuO reinforcement. Figure 6 shows the changes obtained in the mass attenuation coefficients of glass samples at different photon energies, such as 0.05, 0.06, 0.08, 0.1 and 0.15 MeV, respectively. It can be seen in Figure 6 increasing the CuO reinforcement also incredases the mass attenuation coefficients of all glasses from 0 to 8% CuO reinforcement. However, we found that the impact of the increasing CuO reinforcement was more dominant at the lower energies (i.e., 0.5 MeV). This may be explained by the fact that penetration of low-energy gamma rays is more difficult than penetration of moderate- or high-energy photons. It is worth noting that identical patterns were seen for all photon energies examined. The HVL (T_1/2_) is important when determining a material’s shield thickness since it reduces the incident photon intensity to half of its initial value. In this study, the T_1/2_ values of the samples were determined within the same energy range, using attenuation coefficients.

**Figure 7:**
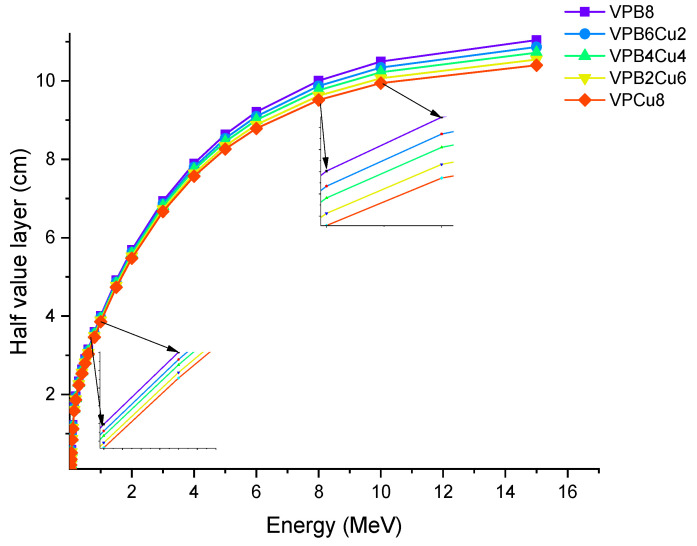
Variation of half value layer (T_1/2_) against photon energy for VPB_x_Cu_y_ glass samples.

**Figure 8:**
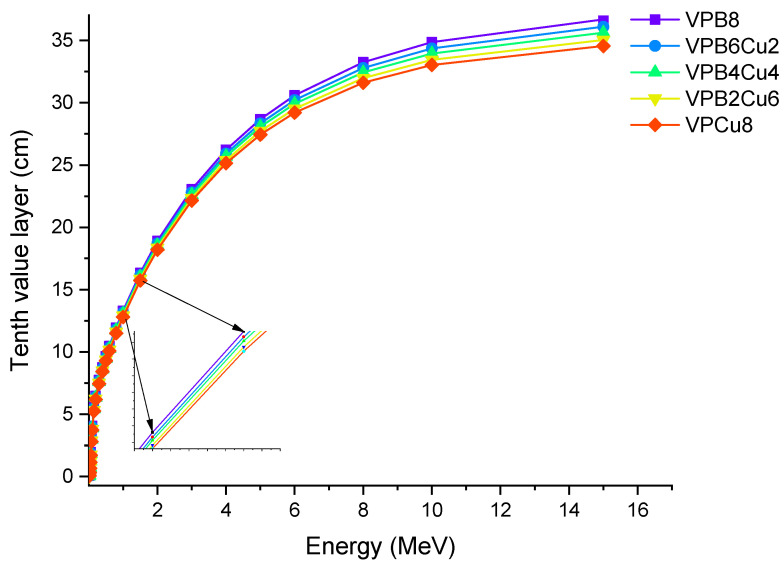
Variation of tenth value layer (T_1/10_) against photon energy for VPB_x_Cu_y_ glass samples.

Figure 7 shows the variation of T_1/2_ as a function of photon energy. With increasing photon energy, we found a direct increase in the glass thickness required, resulting in a 50% reduction in incident photon intensity. Changes in the penetrating qualities of energetic photons as they transition from low to high energy levels can explain this. To put it another way, higher-energy photons will necessitate thicker materials in order to reduce their intensity by half [24]. As discussed earlier, VPCu8 has the highest *μ* values. Therefore, it is worth noting that the HVL and *μ* values have an inverse relationship (see Equation (5)). As a result, higher *μ* values can result in lower T_1/2_ levels, and vice versa. Our findings that the sample VPCu8 had the lowest T_1/2_ values among the studied glass samples verified this fact. At 0.03 MeV, T_1/2_ values of the samples were 0.112, 0.107, 0.102, 0.097 and 0.094 cm for samples VPB8, VPB6Cu2, VPB4Cu4, VPB2Cu6 and VPCu8, respectively. The variance of the tenth value layer as a characteristic of photon energy is depicted in Figure 8.

**Figure 9:**
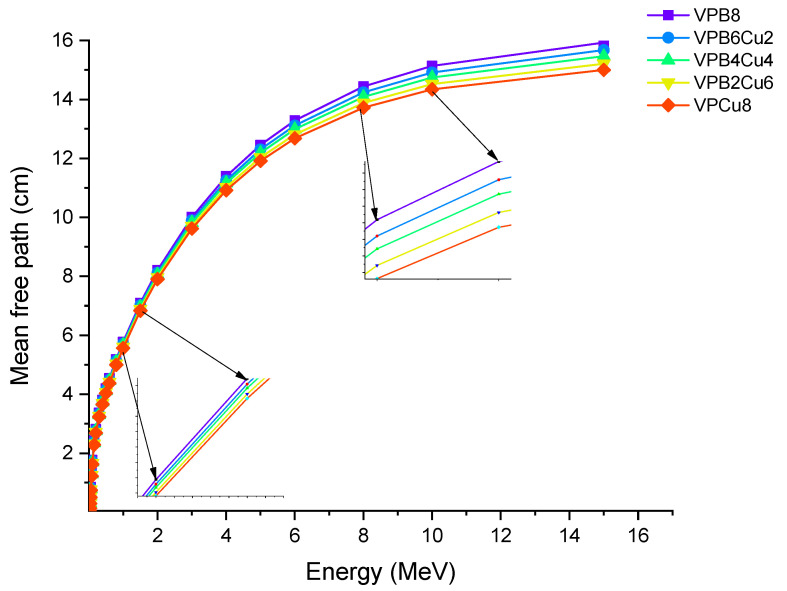
Variation of mean free path (λ) against photon energy for VPB_x_Cu_y_ glass samples.

For the glass samples analyzed, a similar fluctuation pattern was observed with HVL. VPCu8 was likewise found to have the lowest T_1/10_ values. This result confirms the efficiency of the sample in terms of halving the primary radiation intensity and reducing it to one tenth of its original level. MFP is the average distance traveled before an encounter by photons. We calculated the MFP values of the glass samples in terms of their attenuation characteristics as a function of the average distance travelled by an incident photon [25,26]. Figure 9 shows the MFP variation as a function of photon energy for all glass samples. With rising photon energy, the average travel distance of an incoming gamma ray rose. It can be explained by photons’ direct penetration properties, which are affected by their initial energy. The minimum MFP values can be taken as a sign of greater gamma ray attenuation because it indicates that the incident photon’s average traveling distance is also the shortest. The results revealed that sample VPCu8 has the lowest MFP value. This can be explained by the atomic structure and material density of VPCu8, which had a tight atomic structure and a greater density (2.9325 g/cm^3^), preventing the gamma photons from passing through. For instance, at 0.03 MeV the MFP values were 0.162, 0.154, 0.14, 0.141 and 0.135 cm for glass samples VPB8, VPB6Cu2, VPB4Cu4, VPB2Cu6 and VPCu8, respectively.

**Figure 10:**
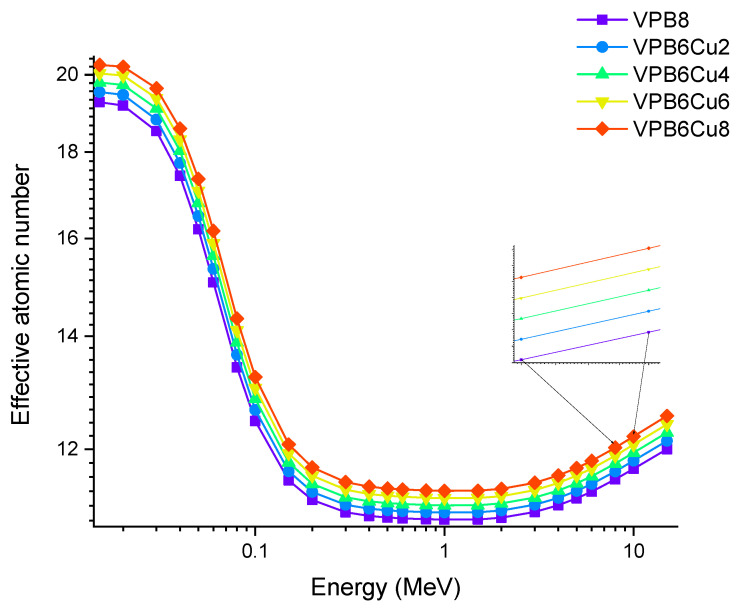
Variation of effective atomic number (Z_eff_) against photon energy for VPB_x_Cu_y_ glass samples.

Figure 10 depicts the change in effective atomic number (Z_eff_) values as a function of photon energy. In general, higher-atomic-number elements are thought to have better gamma ray attenuation. Furthermore, materials having a higher fraction of elements with higher Z values absorb gamma rays more effectively. The obtained results showed that VPCu8 has the highest Zeff value. The maximum difference in Z_eff_ values was seen at 0.04 MeV as 17.43,17.73,18.03,18.31, and 18.58 for glass samples VPB8, VPB6Cu2, VPB4Cu4, VPB2Cu6 and VPCu8, respectively, in the region where photoelectric interactions are predominant. In our study, it was observed that as the % of CuO increases, Z_eff_ values increase proportionally.

**Figure 11:**
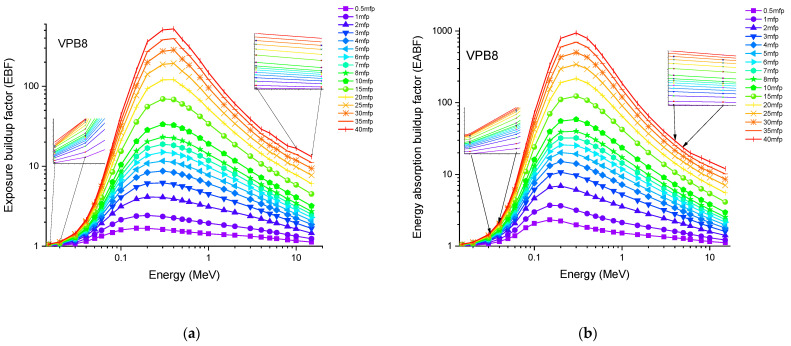
Variation of (**a**) EBF and (**b**) EABF against photon energy for sample VPB8.

**Figure 12:**
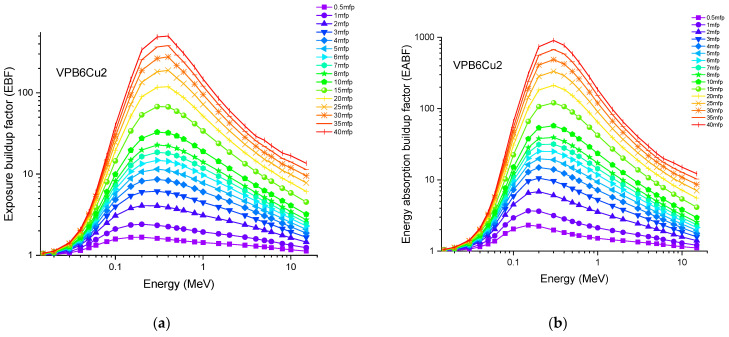
Variation of (**a**) EBF and (**b**) EABF against photon energy for sample VPB6Cu2.

**Figure 13:**
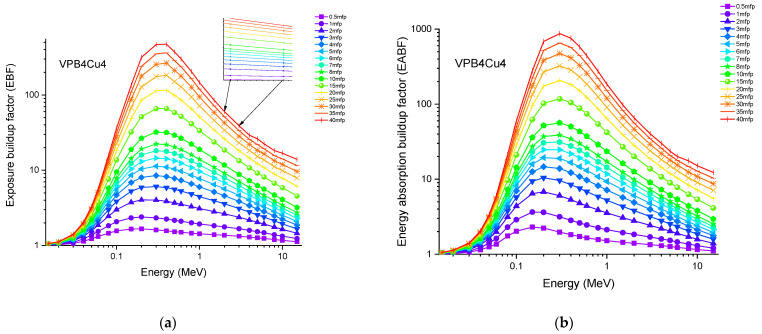
Variation of (**a**) EBF and (**b**) EABF against photon energy for sample VPB4Cu4.

**Figure 14:**
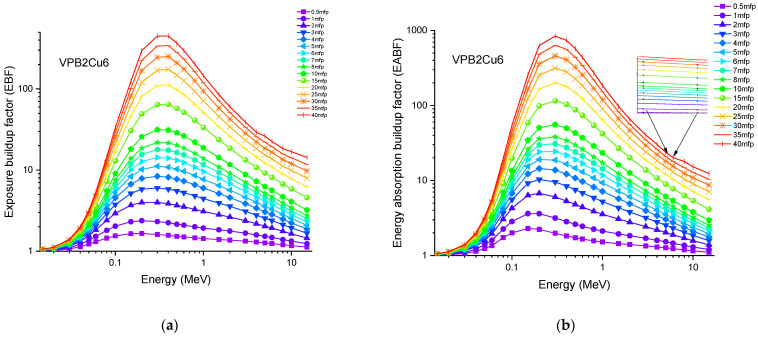
Variation of (**a**) EBF and (**b**) EABF against photon energy for sample VPB2Cu6.

**Figure 15:**
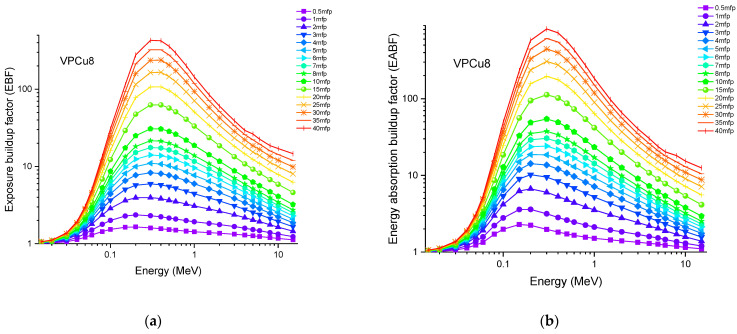
Variation of (**a**) EBF and (**b**) EABF against photon energy for sample VPCu8.

Accordingly, the glass sample with 0% CuO (VPB8) has the lowest Z_eff_ values. The EABF and EBF are significant photon shielding characteristics that are used to define scattering in irradiated materials. The variance in EBF and EABF values for all glass material was analyzed using the geometric progression (G-P) fitting technique for the energy range 0.015 MeV to 15 MeV and penetration depth range of 0.5 to 40, mfp, as shown in Figure 11a,b, Figure 12a,b, Figure 13a,b, Figure 14a,b and Figure 15a,b. It increases with decreasing incoming photon energies until it reaches a maximum in the intermediate energy range, at which point it begins to decline again. The majority of the gamma ray absorption occurs at lower energies, where the photoelectric effect is prominent, and at higher energies, when photoelectric interactions are predominant, according to the literature. The accumulation of photon energy is restricted in these energy zones. For its part, Compton scattering is the most often observed process at intermediate energies because it includes photon absorption rather than absolute photon loss. As a consequence, the EBF values in the Compton area are at their highest levels ever recorded in the region. In this study, EBF values were observed to be the lowest for sample VPCu8. It is worth noting that as the content of CuO in a sample increases, the EBF values decrease. For example, at 0.1 MeV and 40 mfp the EBF values for were 43.622 and 30.634 for samples VBP8 and VPCu8, respectively. The EABF component is a quantity influenced by the amount of energy in the substance as well as the detector characteristic in the interacting material. A variation trend similar to that seen in EBF is observed for EABF. Likewise, the lowest values are reported for the sample with the highest CuO content, VPCu8.

**Figure 16:**
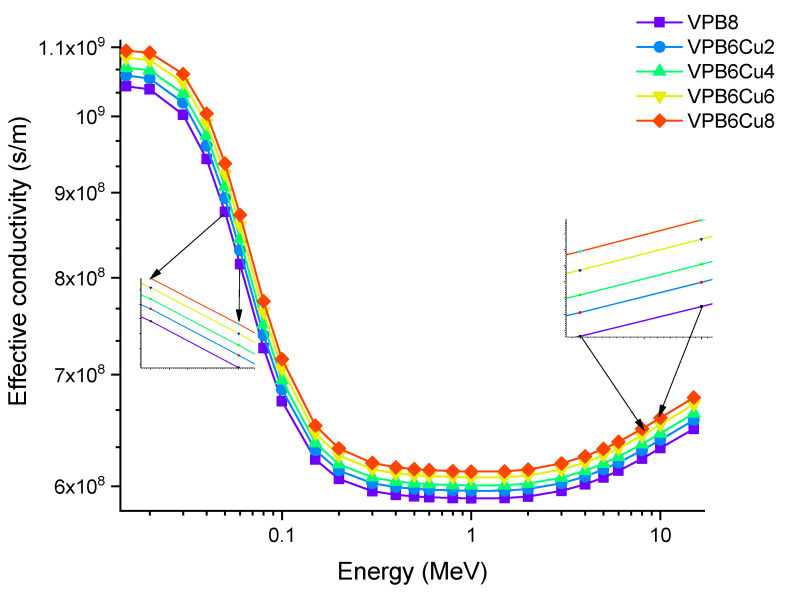
Variation of effective conductivity (C_eff_) at 300K (s/m) for VPB_x_Cu_y_ glass samples.

Finally, Figure 16 depicts the variation in effective conductivity (C_eff_) for all glass samples at 300K (s/m). The effective conductivity C_eff_ (s/m) values of the examined glass samples were obtained in the photon energy range of 0.015–15 MeV. The effective conductivity C_eff_ (s/m) of a shielding-material for attenuation at room temperature (300 K) is proportional to the effective number of electrons per gram. As observed in Figure 16, C_eff_ has an inverse relationship with photon energy, that is, as the energy increases, C_eff_ values decrease rapidly, especially in the region where photoelectric interactions are dominant [27]. Furthermore, the sample with the highest CuO content (VPCu8) has the highest C_eff_ values at all photon energies.

Finally, the HVL values for the VPCu8 sample were compared to those for various kinds of concrete [28] and the ZBV4 sample [29]. The HVL values obtained for comparable materials in the 0.015–15 MeV photon energy range are shown in Table 3. Our results indicate that the superior VPCu8 sample has lower HVL values than ordinary concrete (OC) at the photon energies investigated. One might argue that the VPCu8 sample is a good candidate for usage in radiation facilities where OC serves as the primary shield protecting personnel and public health. In comparison to the VPCu8 sample, basalt-magnetite concrete and hematite-serpentine concrete were found to have somewhat lower HVL values. It can be inferred that these additional improvements may be made to the VPCu8 sample’s current gamma ray attenuation capabilities in order to further decrease the HVL values. Increasing CuO reinforcement may be regarded as an enhancement approach for this purpose. However, it is important to explore the impact of additional reinforcement values on optical, structural, and mechanical characteristics on behavioral assessment, since these qualities may potentially affect the appropriateness of the VPCu8 sample for shielding purposes.

## 4. Conclusions

This study aimed to determine the nuclear radiation shielding abilities of five different CuO-doped vanadate glasses from the 46V_2_O_5_-46P_2_O_5_-(8-x) B_2_O_3_-xCuO in a wide range of incident photon energies between 0.015 and 15 MeV by using both the Phy-x PSD platform and MCNPX Monte Carlo code. An initial conclusion to be noted is that the increase in the % of CuO in the sample (0 to 8 mol%) leads to a linear increase in the density of the samples from 2.8123 to 2.9325 g/cm^3^. Moreover, the sample with the highest Cu content showed the greatest attenuation of gamma rays, indicating that CuO reinforcement has a potent anti-nuclear radiation property. The outcomes of this study can be further summarized as follows, the *μ*_m_ values at 0.100 MeV were 0.201, 0.203, 0.206, 0.208 and 0.211 cm^2^/g for glass samples VPB8, VPB6Cu2, VPB4Cu4, VPB2Cu6, and VPCu8, respectively, revealing that VPCu8 has the highest values. Furthermore, the findings of our study showed that sample VPCu8 has the lowest T_1/2_, T_1/10,_ and λ at all the photon energies investigated. Moreover, sample VPCu8 reported the highest Z_eff_ and C_eff_ values. In regard to EBF and EABF, the maximum values were reported for the sample with 0% CuO (i.e., VPB8), and the lowest values were reported for VPCu8. Accordingly, we were able to determine that the VPCu8 glass sample has superior nuclear shielding abilities, which proves that the reinforcement of CuO in vanadate glasses provides.

## Figures and Tables

**Table 1:** Glass sample codes, composition, elemental weight fractions, and densities (wt.%).

Sample Code	B	O	P	V	Cu	Density (g/cm^3^)
VPB8	0.011193	0.501109	0.184408	0.30329	0	2.8123 ± 0.0001
VPB6Cu2	0.008384	0.49633	0.184171	0.302901	0.008214	2.8429 ± 0.0001
VPB4Cu4	0.005582	0.491563	0.183935	0.302513	0.016407	2.8656 ± 0.0001
VPB2Cu6	0.002787	0.486809	0.1837	0.302125	0.024579	2.8984 ± 0.0001
VPCu8	0	0.482066	0.183465	0.301739	0.03273	2.9235 ± 0.0001

**Table 2:** The obtained mass attenuation coefficients (*μ*_m_) of VPB_x_Cu_y_ glasses from Phy-X/PSD program and MCNPX (v. 2.7.0) code along with % deviations.

	VPB8			VPB6Cu2			VPB4Cu4			VPB2Cu6			VPCu8		
	Phy-X/PSD	MCNPX	∆ (%) *	Phy-X/PSD	MCNPX	∆ (%) *	Phy-X/PSD	MCNPX	∆ (%)*	Phy-X/PSD	MCNPX	∆ (%) *	Phy-X/PSD	MCNPX	∆ (%) *
0.015	15.287	15.456	0.27	15.867	16.045	0.28	16.445	16.961	0.77	17.022	17.238	0.32	17.597	17.788	0.27
0.02	6.786	6.814	0.10	7.050	7.141	0.32	7.314	7.547	0.78	7.577	7.713	0.44	7.840	7.912	0.23
0.03	2.193	2.231	0.43	2.277	2.346	0.75	2.362	2.563	2.04	2.446	2.563	1.17	2.530	2.614	0.82
0.04	1.031	1.101	1.64	1.068	1.126	1.32	1.105	1.132	0.60	1.141	1.158	0.37	1.178	1.217	0.81
0.05	0.608	0.612	0.16	0.627	0.634	0.28	0.647	0.652	0.19	0.666	0.674	0.30	0.685	0.701	0.58
0.06	0.418	0.421	0.18	0.429	0.432	0.17	0.440	0.456	0.89	0.452	0.472	1.08	0.463	0.479	0.85
0.08	0.261	0.263	0.19	0.266	0.271	0.47	0.271	0.289	1.61	0.276	0.292	1.41	0.280	0.292	1.05
0.1	0.201	0.204	0.37	0.203	0.209	0.73	0.206	0.211	0.60	0.208	0.214	0.71	0.211	0.225	1.61
0.15	0.147	0.149	0.34	0.148	0.151	0.50	0.148	0.154	0.99	0.149	0.158	1.47	0.150	0.159	1.46
0.2	0.126	0.128	0.39	0.127	0.129	0.39	0.127	0.134	1.34	0.127	0.137	1.89	0.127	0.137	1.89
0.3	0.106	0.112	1.38	0.106	0.114	1.82	0.106	0.117	2.47	0.106	0.119	2.89	0.106	0.119	2.89
0.4	0.093	0.101	2.06	0.093	0.104	2.79	0.093	0.107	3.50	0.093	0.109	3.96	0.093	0.109	3.96
0.5	0.085	0.092	1.98	0.085	0.093	2.25	0.085	0.095	2.78	0.085	0.097	3.30	0.085	0.098	3.55
0.6	0.078	0.084	1.85	0.078	0.085	2.15	0.078	0.087	2.73	0.078	0.089	3.29	0.078	0.091	3.85
0.8	0.069	0.073	1.41	0.069	0.073	1.41	0.069	0.071	0.71	0.068	0.073	1.77	0.068	0.071	1.08
1	0.062	0.065	1.18	0.062	0.065	1.18	0.062	0.065	1.18	0.061	0.065	1.59	0.061	0.065	1.59
1.5	0.050	0.054	1.92	0.050	0.054	1.92	0.050	0.054	1.92	0.050	0.054	1.92	0.050	0.054	1.92
2	0.043	0.047	2.22	0.043	0.048	2.75	0.043	0.049	3.26	0.043	0.049	3.26	0.043	0.049	3.26
3	0.036	0.039	2.00	0.036	0.039	2.00	0.036	0.039	2.00	0.036	0.04	2.63	0.036	0.039	2.00
4	0.031	0.033	1.56	0.031	0.033	1.56	0.031	0.033	1.56	0.031	0.033	1.56	0.031	0.034	2.31
5	0.029	0.031	1.67	0.029	0.031	1.67	0.029	0.031	1.67	0.029	0.031	1.67	0.029	0.031	1.67
6	0.027	0.029	1.79	0.027	0.031	3.45	0.027	0.031	3.45	0.027	0.032	4.24	0.027	0.031	3.45
8	0.025	0.026	0.98	0.025	0.026	0.98	0.025	0.026	0.98	0.025	0.026	0.98	0.025	0.026	0.98
10	0.023	0.025	2.08	0.024	0.025	1.02	0.024	0.025	1.02	0.024	0.025	1.02	0.024	0.025	1.02
15	0.022	0.024	2.17	0.022	0.024	2.17	0.023	0.025	2.08	0.023	0.025	2.08	0.023	0.026	3.06

* $∆\;\left(\%\right)=\frac{\left|\mu_{m}\left(\mathrm{MCNPX}\right)-\mu_{m}\left(\mathrm{Phy-X/PSD}\right)\right|}{\left[\mu_{m}\left(\mathrm{MCNPX}\right)+\mu_{m}\left(\mathrm{Phy-X/PSD}\right)\right]/2}\times 100$

**Table 3:** Comparison of HVL values for VPCu8 sample with different types of concretes and ZBV4 sample.

Photon Energy	Current Study	[28]				[29]
E (MeV)	VPCu8	Ordinary Concrete	Basalt-Magnetite Concrete	Hematite-Serpentine Concrete	Steel-Scrap Concrete	ZBV4
0.015	0.01347	0.04339	0.01127	0.01311	0.00686	0.00513
0.02	0.03024	0.10046	0.02543	0.0295	0.01546	0.01121
0.03	0.09371	0.30814	0.08033	0.09301	0.04917	0.03434
0.04	0.20123	0.60803	0.17672	0.20492	0.07489	0.07575
0.05	0.34606	0.92174	0.31196	0.3629	0.1383	0.13761
0.06	0.51233	1.19271	0.47238	0.5522	0.22165	0.21891
0.08	0.84554	1.57142	0.8039	0.94984	0.42787	0.42438
0.10	1.12456	1.80291	1.08375	1.29198	0.64371	0.65166
0.15	1.58187	2.14202	1.52729	1.84225	1.06051	1.14313
0.20	1.86052	2.37485	1.78384	2.16102	1.31577	1.47907
0.30	2.24172	2.74889	2.12792	2.58396	1.63017	1.90543
0.40	2.53800	3.0715	2.39752	2.9133	1.85433	2.20374
0.50	2.79604	3.36401	2.63369	3.20123	2.04516	2.45004
0.60	3.03274	3.6365	2.8511	3.46617	2.21793	2.66951
0.80	3.46298	4.13916	3.24891	3.95012	2.53158	3.062
1.00	3.85691	4.60294	3.61478	4.39535	2.81859	3.41741
1.50	4.73592	5.64834	4.43264	5.38995	3.45262	4.19939
2.00	5.47754	6.54876	5.12196	6.22634	3.96992	4.84036
3.00	6.66721	8.04572	6.22293	7.56504	4.75149	5.82327
4.00	7.56844	9.23181	7.05124	8.57325	5.2912	6.52275
5.00	8.25911	10.19199	7.68294	9.33846	5.67038	7.02483
6.00	8.78893	10.96492	8.16019	9.92337	5.93042	7.38462
8.00	9.51359	12.10822	8.80858	10.71325	6.22662	7.81974
10.00	9.94309	12.87456	9.1897	11.17529	6.35216	8.02951
15.00	10.39940	13.89992	9.57293	11.63975	6.36616	8.13915

## Data Availability

The data presented in this study are available on request from the corresponding author.
